# Effect of Fractioning on Antibacterial Activity of* Enantia chlorantha* Oliver (Annonaceae) Methanol Extract and Mode of Action

**DOI:** 10.1155/2018/4831593

**Published:** 2018-04-29

**Authors:** Rebeca Ebelle Etame, Raymond Simplice Mouokeu, Cedric Laurel Cidjeu Pouaha, Igor Voukeng Kenfack, Raphael Tchientcheu, Jean Paul Assam Assam, Frank Stève Monthe Poundeu, Alembert Tchinda Tiabou, François Xavier Etoa, Jules Roger Kuiate, Rosalie Anne Ngono Ngane

**Affiliations:** ^1^Institute of Medical Research and Medicinal Plant Studies (IMPM), P.O. Box 6163, Yaoundé, Cameroon; ^2^Institute of Fisheries and Aquatic Sciences, University of Douala, P.O. Box 7236, Douala, Cameroon; ^3^Faculty of Sciences, University of Douala, P.O. Box 24157, Douala, Cameroon; ^4^Faculty of Sciences, University of Dschang, P.O. Box 67, Dschang, Cameroon; ^5^Faculty of Sciences, University of Yaoundé I, P.O. Box 812, Yaoundé, Cameroon

## Abstract

Infectious diseases caused by bacteria constitute the main cause of morbidity and mortality throughout the world and mainly in developing countries. In this work, the influence of fractioning and the mode of action of stem barks methanol extract of* Enantia chlorantha* were investigated. The aim was to optimize the antibacterial activity of the methanol extract. The extract was prepared by maceration of barks powder in methanol. Fractioning was done using increasing solvents polarity. Standard phytochemical methods were used for phytochemical screening. Minimum Inhibitory Concentrations (MIC) and Minimum Bactericidal Concentration (MBC) of the methanol extract and fractions were determined using broth microdilution method. The studied mode of action of both methanol extract and n-butanol fraction included antibiofilm activity, H+-ATPase-mediated proton pumping assay, salt tolerance, and cells cycle. The methanol extract of* E. chlorantha* stem barks was found to be active on all the bacteria tested (32 ≤ MIC ≤ 512 *μ*g/mL), its activity being significant (MIC < 100 *μ*g/ml) out of 5 of the 28 clinical isolates used.* Salmonella enterica *serovar paratyphi A was the most sensitive (32 *μ*g/mL). Compared to the extract and other fractions, the n-butanol fraction was found to be more active (32 ≤ MIC ≤ 256). Significant antibacterial activity of this fraction was observed out of 10 of the 28 bacterial isolates and 3 out of 7 bacterial strains. Lowest MIC values (32 *μ*g/ml) of this fraction were obtained with* Escherichia coli* (136),* Pseudomonas aeruginosa* (CIP 76110), and* Salmonella enterica* serovar typhi 9. The methanol extract of* E. chlorantha* and its n-butanol fraction revealed several modes of action including the prolongation of the latency phase of the bacterial growth, the inhibition of the pump with protons H^+^ - ATPases bacterial, the loss of the salt tolerance of the* Staphylococcus aureus,* and inhibition of the formation of the bacterial biofilm. The present results showed that the n-butanol fraction of the methanol stem barks extract of* E. chlorantha* possess the essential antibacterial components and could best be used to fight against bacterial infections as compared to methanol extract.

## 1. Introduction

Infectious diseases caused by bacteria constitute a public health problem all over the world and particularly in Africa where living conditions are precarious [1, 2]. The discovery of antibiotics has been a real relief for humanity because they have greatly reduced the incidence of infectious diseases [3]. The often inappropriate prescription of antibiotics and their inappropriate use by the population among others have led to selection pressure by bacteria [4]. This has led, for several decades, to the emergence of resistant bacteria, which is the main cause of increased frequency of therapeutic failures, mortality, and high costs of treatment [5]. Alternative remediation could include new antimicrobial substances that are effective, available, and low in toxicity. Medicinal plants are a credible research pathway. Indeed, they are rich in molecules of an exceptional chemical variability making them a vast reservoir of substances that can act through various modes of action [6]. Many approaches have so far been used to demonstrate the antibacterial activity of plants. They include crude extract preparation using variety of solvents [7], purification of active compounds from extracts [8–11], successive extraction with solvents with increasing polarity [12], and distillation. Solvent extraction has been the most used method but recent studies established increased activity with fractionation of crude extract [13, 14]. In addition, many African countries with poor technology are limited for production of medicine from pure active compounds or their derivatives. Alternative could be formulation of best active plant extract or their fractions.

*Enantia chlorantha* is a plant belonging to the Annonaceae family. It is highly prized in the traditional Cameroonian pharmacopoeia and used in the treatment of several infections including malaria, anemia, typhoid fever, and yellow fever [15]. Also called Epoue (Baka), Peye (Badjoue), and Nfol (Bulu), it is widely spread along Sub-Saharan Africa [16]. The stem barks, leaves, and roots are used in Africa to treat jaundice, urinary infection, and leprosy spots. They are also used as hemostatic agents and uterus stimulants [17]. Previous studies highlighted antimicrobial, antimycobacterial, antiviral, antioxidant, antipyretic, and antisickin activity [18]. The present work was initiated to improve the antibacterial activity of the methanol extract and to explore possible mechanism of bactericidal action.

## 2. Materials and Methods

### 2.1. Materials

#### 2.1.1. Plant Material

The barks of* E. chlorantha* were collected at Kalla Mountain, Central Region of Cameroon. The plant was identified at the National Herbarium (Yaoundé, Cameroon) where a voucher specimen was deposited under the reference number 45569/HNC.

#### 2.1.2. Chemicals

Chloramphenicol (Sigma-Aldrich, St. Quentin Fallavier, France) was used as reference antibiotic.* p*-Iodonitrotetrazolium chloride (INT) was used as microbial growth indicator.

#### 2.1.3. Bacteria Strains, Clinical Isolates, and Culture Media

The antibacterial activities of crude extract and fractions were carried out on 7 strains and 28 clinical isolates. The clinical isolates of* Escherichia coli*,* Enterobacter aerogenes*,* Klebsiella pneumoniae,* and* Staphylococcus aureus* were obtained from patient suffering from the gastroenteritis at the Bafang ADLUCEM hospital. The isolate of* Salmonella enterica serovar typhi*,* Salmonella enterica serovar paratyphi *A,* Salmonella enterica serovar paratyphi* B, and* Salmonella enterica serovar typhimurium* were obtained from the Laboratory of Bacteriology and Mycology of the “Centre Pasteur” Yaoundé-Cameroon. Methicillin-resistant* Staphylococcus aureus* strains were obtained from the culture collection of the Laboratory of Microbiology, Graduate School of Pharmaceutical Sciences, University of Tokyo, Japan. Multidrug resistant* Providencia stuartii* strain was obtained from the culture collection of the University of Mediterranean, France. The bacterial features are summarized in Table 1 [19–22].

These microorganisms were maintained at 4°C on Mueller Hinton Agar (MHA) (Liofilchem, Italy). Mueller Hinton Broth (MHB) (Liofilchem, Italy) was used for Minimum Inhibitory Concentrations (MIC) and Minimum Bactericidal Concentrations (MBC) determination.

### 2.2. Methods

#### 2.2.1. Extract Preparation and Fractioning

*E. chlorantha* barks were dried for 14 days and powdered. 1 kg dry powder was soaked in 5 L methanol for 48 hours at room temperature and then filtered using Whatman filter paper number 1. The filtrate obtained was concentrated at 45°C under reduced pressure in a vacuum to obtain plant extract (7.91%).

Fractionation of the methanol extract of dry powder of* E. chlorantha* barks was done using successive partitions. In so doing, 80 g methanol extract was dissolved in 300 mL distilled water and shacked vigorously in the presence of 500 mL hexane and was allowed to decant. The hexane phase was further collected and the process repeated twice. The same process was repeated with ethyl acetate and n-butanol and the hexane, ethyl acetate, and n-butanol phases were concentrated at 45°C under reduced pressure in a Rota vapor. The residual fraction was dried for two days at 45°C in a stove.

#### 2.2.2. *In Vitro *Antibacterial Activity

*(a) Preparation and Standardization of Inocula*. The inoculum of each bacterium was prepared by dilution in distilled water, three to four colonies obtained from 18 hours culture on MHA at 37°C. These microbial suspensions were diluted to match the 0.13 optical density at 600 nm corresponding to 1.5 × 10^6^ CFU/mL using a Genesys 20 UV/Vis spectrophotometer.

*(b) INT Colorimetric Assay for Minimum Inhibitory Concentrations (MIC) and Minimum Bactericidal Concentrations (MBC) Determination*. The inhibitory potential of bacteria growth by methanol extract and fractions from* E. chlorantha* was determined by broth microdilution method in 96-well microtiter plates [23]. The MIC assay was conducted using INT colorimetric assay according to Eloff [24] with slight modifications. Test samples or chloramphenicol was dissolved in dimethylsulfoxide (DMSO). A serial twofold dilution was performed to obtain final concentrations ranging from 16 to 1024 *μ*g/mL. Each well was further diluted with 100 *μ*l inoculum. The plates were incubated at 37°C for 18 h. The assay was repeated thrice. Wells containing adequate broth, bacterial inoculum, and DMSO to a final concentration of 2.5% served as negative control. Following incubation, 40 *μ*L of 0.2 mg/mL INT was added in each well. Plates were further incubated for additional 30 min at 37°C. Viable bacteria reduced the yellow dye of INT to a pink color. MIC values were defined as the sample concentration that prevented the color change of INT that exhibited complete inhibition of bacterial growth [25].

The MBC values were determined by adding 50 *μ*l aliquots of the preparations which did not show any growth after incubation during MIC assays to 150 *μ*L broth culture medium. These preparations were incubated at 37 °C for 48 h. The MBC values were regarded as the lowest concentration of extracts which did not produce any color change after addition of INT as mentioned above [26]. The tests were done in triplicate and repeated thrice.

#### 2.2.3. Antibacterial Mechanisms Study

*(a) Bacteria Cell Growth*. The effect of methanol extract and n-butanol fraction from* E. chlorantha* on* E. coli* and* S. aureus *cell growth was performed according to Darah et al. [27] with some modifications. Bacterial suspensions were prepared as described previously. Extract or n-butanol fraction was added to conical plates containing 23 mL of sterilized Mueller Hinton Broth culture medium and inoculum (1.5 × 10^6^ CFU/mL). The final concentrations of the extracts in the plates were half MIC, MIC, and 2 MIC. Plates containing only culture media and those containing culture media and bacteria suspension constitute negative control, while plates containing chloramphenicol at MIC were considered as positive control. The experiments were performed in triplicate and all the plates were incubated at 37°C under agitation at 150 rpm. After the incubation period corresponding to *t* = 0 min, 30 min, 1 h, 2 h, 4 h, 6 h, 8 h, 10 h, 12 h, 14 h, 16 h, and 18 h, the optical density of each flask was recorded at 600 nm using BIOBASE UV-VIS spectrophotometer. All the measurements were done in triplicate.

*(b) H* ^+^ *-ATPase-Mediated Proton Pumping*. The proton pumping activities of* E. coli* and* S. aureus* were determined by monitoring glucose-induced acidification of the external medium [28]. Briefly, 100 mL bacteria culture was grown in Muller Hinton broth culture for 18 hours at 37°C. The resulting culture was centrifuged at 3500 g for 10 min. The pellet was washed twice with distilled water and then with 50 mM KCl and resuspended in 50 mL of 50 mM KCl. The cell suspension was incubated overnight (18 h) at 4°C for glucose starvation and then centrifuged and diluted to achieve absorbance of 8 at 600 nm. In 4 mL of the reaction medium, 0.5 mL methanol extract of* E. chlorantha *or n-butanol fraction at half MIC, MIC, and 2 MIC were added and the pH adjusted to 6.4. Upon 10 min preincubation at 37°C, medium acidification was initiated with addition of glucose 20% (0.5 mL) followed by pH measurement after each 10 min for 2 h. DMSO 2.5% was used as control. The experiment was performed in triplicate and repeated thrice.

*(c) Loss of Salt Tolerance in Staphylococcus aureus*. The ability of* S. aureus* ATCC 25923 and* S. aureus* ST 120 cells treated with methanol extract and n-butanol fraction to grow on Mueller Hinton agar (MHA) supplemented with NaCl was investigated. In preliminary experiments, untreated suspensions of* S. aureus *were plated on MHA supplemented with NaCl from 40 to 100 mg/mL. Plates were incubated and upon incubation, the resulting colonies were counted. Concentrations of NaCl, 60, 70, and 75 mg/mL, that modestly compromised the colony-forming abilities were selected. For further experimentation steps, suspensions of bacteria were prepared as described previously and treated with extract of* E. chlorantha *and n-butanol fraction at half MIC, MIC, and 2 MIC. After 1 h incubation period, samples were inoculated on MHA plates supplemented with NaCl (60, 70, and 75 mg/mL). Bacterial culture without extract was used as control for each MHA-NaCl plate. Upon incubation, the mean number of colony-forming units/mL on agar medium was reported [29].

*(d) Biofilm Formation*. Biofilm assays were conducted based on the method described by O'Toole and Kolter [30] with slight modifications. Bacteria cell suspensions were prepared as described previously. In sterile 96-well culture plates, containing 100 *μ*L of Mueller Hinton Broth, 100 *μ*L extract or n-butanol fraction solution was added in the first well. A serial twofold dilution was performed to obtain final concentration range of 1/2 MIC to 8 MIC. 100 *μ*L of fresh bacterial suspension was added. After 24 h incubation at 37°C, the content of each well was gently removed by tapping the plates. The wells were washed with 200 *μ*L sterile saline to remove free-floating bacteria and then dried and fixed at 65°C for 1 h. Biofilms formed by adherent cells in plates were stained with 0.8% crystal violet and incubated at room temperature for 20 minutes. Excess stains were rinsed off by thorough washing with deionized water and plates were fixed with 200 *μ*L of 30% acetic acid. Growth control (cells + broth), media control (only broth), and blank control (broth + extract) were included in the assay. Optical densities (OD) of stained adherent bacteria were measured at 630 nm using an ELISA microplate reader. The percentage of biofilm inhibition was calculated using the following formula: [(OD growth control − OD sample)/OD growth control] × 100 [31]. All tests were performed in triplicate and repeated twice.

#### 2.2.4. Phytochemical Screening of Methanol Extract and Fractions

The presence of alkaloids, triterpenes, sterols, flavonoids, polyphenols, and saponins was screened according to the common phytochemical methods described by Harbone [32].

#### 2.2.5. Statistical Analysis

The statistical analysis was performed using Statistical Package for the Social Sciences (SPSS) version 20.0 (SPSS, Inc., Chicago, IL, USA). One-way ANOVA and Duncan's multiple range tests were done and the results were considered significant at probability *p* < 0.05.

## 3. Results and Discussion

### 3.1. Antibacterial Activity

The methanol extract and fractions from stem bark of* E. chlorantha* were evaluated for their antibacterial activities on a panel of bacteria including strains (7) and clinical isolates (28). The results are reported in Table 2.

The methanol extract of this plant was active on all the strains and isolates tested with MIC values ranging from 32 to 512 *μ*g/mL. Significant activity was obtained with 5/25 clinical isolates used. The lowest value of 32 *μ*g/mL was obtained with* S. enterica serovar paratyphi* A. Moreover, antibacterial activity of this extract was obtained on multiresistant* S. enterica serovar paratyphi *isolates*, S. enterica serovar typhimurium,* and* S. aureus*.

The fractionation of crude extract of* E. chlorantha* made it possible to obtain four fractions with varying degrees of antibacterial activity (Table 2). n-Hexane and ethyl acetate fractions were found to be less active with MIC < 1024 *μ*g/mL obtained on 29/35 and 30/35 35 tested bacteria, respectively. Compared to these fractions, the n-butanol and residual fractions were most active with MIC values ranging from 32 *μ*g/mL to 512 *μ*g/mL. Significant activity (MIC < 100 *μ*g/mL) was obtained with 13/35 and 7/35 bacteria, respectively, with n-butanol and residual fractions. Lowest MIC (64 *μ*g/mL) was obtained with n-butanol fraction on* S. enterica serovar typhi* (SAL 9),* P. aeruginosa* (CIP 76110), and* E. coli* (EC 136).

### 3.2. Mechanism of Antibacterial Activity

#### 3.2.1. Inhibition of Bacteria Cell Growth

**Figure 1:**
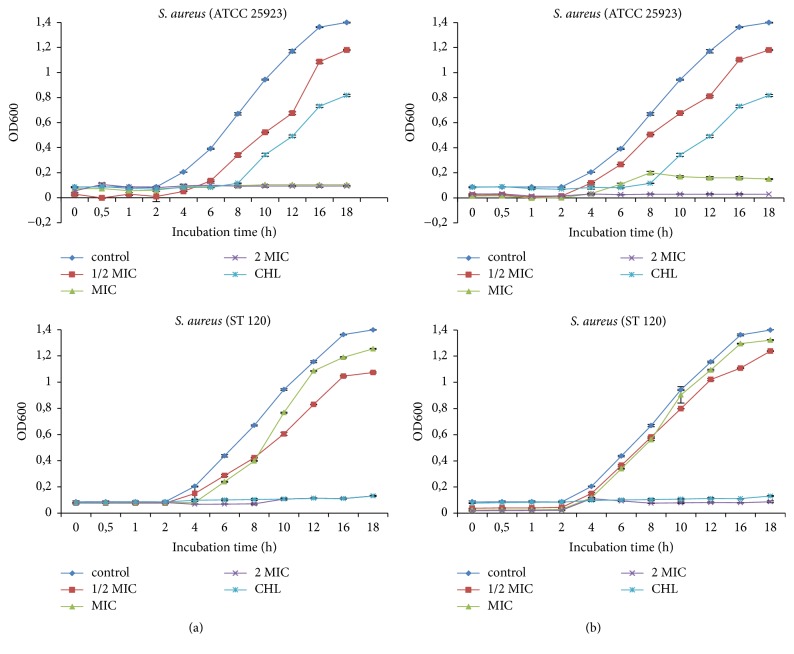
Effect of* E. chlorantha* methanol extract (a) and its n-butanol fraction (b) on* Staphylococcus aureus* growth as a function of time. CHL: chloramphenicol; MIC: minimum inhibitory concentration. Data are expressed as mea, *n* = 3.

**Figure 2:**
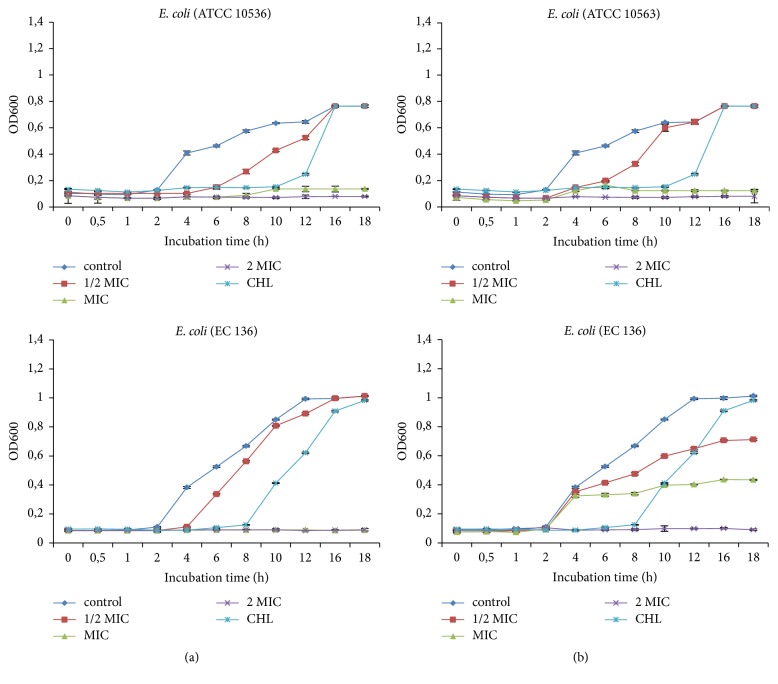
Effect of* E. chlorantha* methanol extract (a) and its n-butanol fraction (b) on* Escherichia coli* growth as a function of time. CHL: chloramphenicol; MIC: minimum inhibitory concentration. Data are expressed as mean; *n* = 3.

Figures 1-2 showed the influence of extracts on the growth curves of* S. aureus* and* E. coli*. In general, the* E. chlorantha* extract and fraction inhibited bacterial cell growth depending on the concentrations and microorganisms. At 1/2 MIC, inhibition of bacterial cell growth was observed after 4 to 6 hours following treatment. At MIC and 2 MIC, inhibition was maximal with prolongation of the latency phase. Furthermore, at these concentrations, both extract and fraction produced cell eradication after 12 h.

#### 3.2.2. Inhibition of H+-ATPase-Mediated Proton Pumping

**Figure 3:**
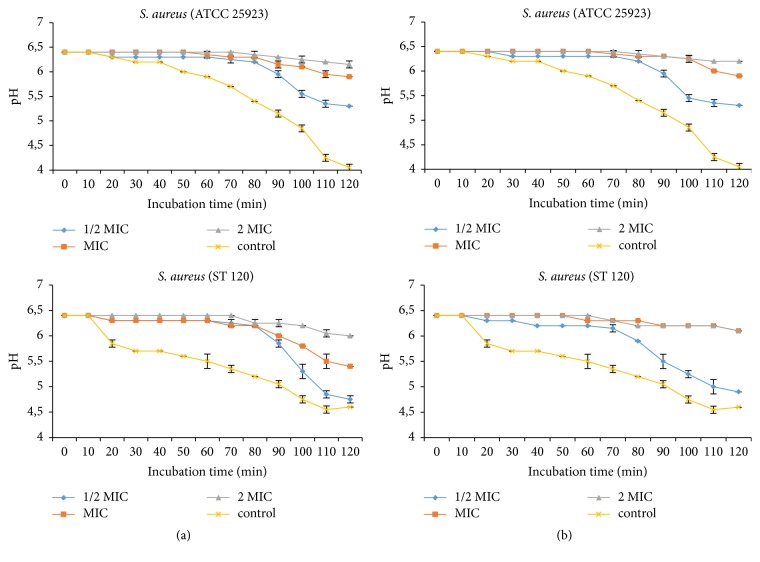
Effect of* E. chlorantha* methanol extract (a) and its n-butanol fraction (b) on* Staphylococcus aureus* proton pumping. MIC: minimum inhibitory concentration; data are expressed as mean ± SEM *n* = 3; Ph was measured after 10 min preincubation.

**Figure 4:**
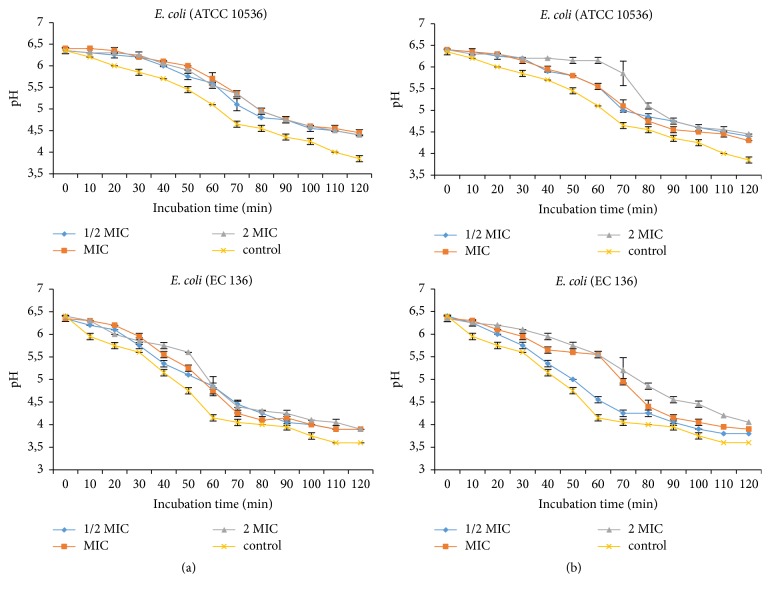
Effect of* E. chlorantha* methanol extract (a) and its n-butanol fraction (b) on* Escherichia coli* proton pumping. MIC: minimum inhibitory concentration; data are expressed as mean ± SEM *n* = 3; Ph was measured after 10 min preincubation.

The methanol extract of* E. chlorantha* bark and its n-butanol fraction independently of the concentration inhibited the bacterial H + -ATPase proton pumps (Figures 3-4). This inhibitory effect was more marked on* E. coli*. In general, the inhibitory effect of the H + -ATPases proton pumps was more pronounced at 2 MIC values.

#### 3.2.3. Loss of Salt Tolerance Capacity

**Figure 5:**
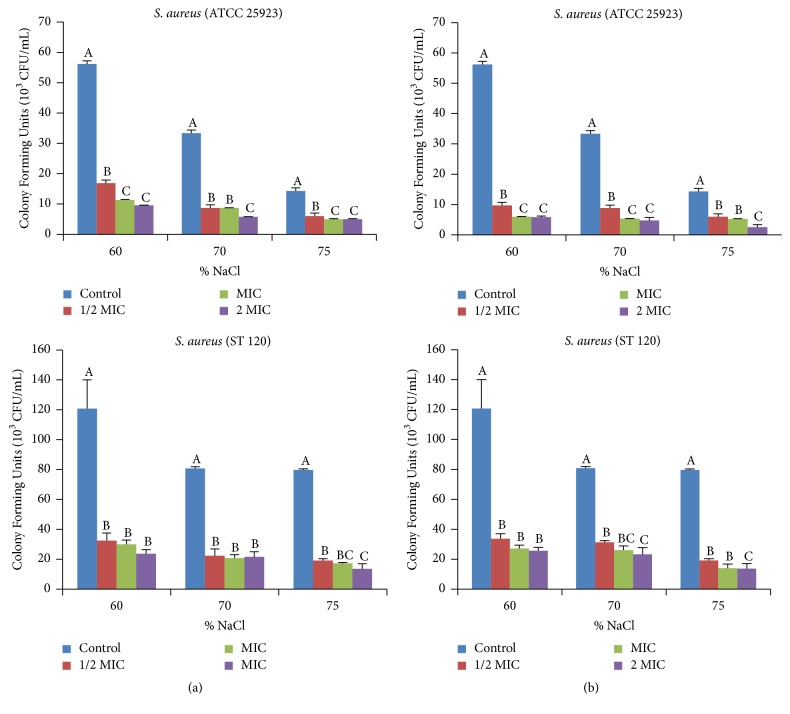
Effect of* E. chlorantha* methanol extract (a) and its n-butanol fraction (b) on the reduction of salt tolerance of* Staphylococcus aureus*. MIC: minimum inhibitory concentration; data are expressed as mean ± SEM *n* = 3. Values with different superscript letters are significantly different at *p* < 0.05 according to Waller Duncan.

The salt tolerance potential of* S. aureus* in the presence of* E. chlorantha* extract or n-butanol fraction at different concentrations is shown in Figure 5. It can be observed that when the bacteria pretreated with extract were inoculated on culture media supplemented with different concentrations of NaCl, a significant decrease in the number of colony-forming units was observed. Compared to other concentrations, the number of colonies formed on culture medium supplemented at 75% NaCl was almost zero at 2 MIC.

#### 3.2.4. Antibiofilm Activity

**Figure 6:**
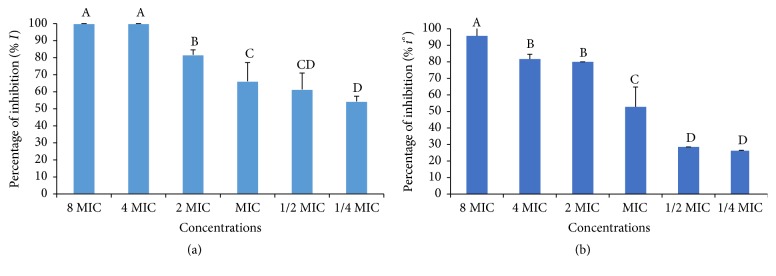
Antibiofilm effect of* E. chlorantha* methanol extract (a) and its n-butanol fraction (b) against* Escherichia coli* (EC 136). MIC: minimum inhibitory concentration; data are expressed as mean ± SEM *n* = 3. Values with different superscript letters are significantly different at *p* < 0.05 according to Waller Duncan.

The extract and the n-butanol fraction have differently inhibited the* E. coli* biofilm formation (Figure 6). The inhibition was maximal (100%) at 8 and 4 MIC with crude extract. The n-butanol fraction was unable to inhibit all of the bacterial biofilm at 8 MIC.

#### 3.2.5. Phytochemical Composition

The qualitative phytochemical composition showed that the methanol extract of* E. chlorantha* bark has at least seven classes of phytochemicals (Table 3). Alkaloids and triterpenes were found almost in all fractions, the other classes of secondary metabolites being selectively distributed. The n-butanol fraction was more rich in secondary metabolites in the same way as the crude extract followed by the residual fraction and the ethyl acetate fraction, respectively.

## 4. Discussion

The resistance of bacteria to usual antibiotics has led to a high incidence of treatment failures and a considerable increase in treatment costs [4]. Medicinal plants could be used as an alternative as their pharmacological properties including antimicrobial activity are well known [33].

The methanol extract of the* E. chlorantha* barks revealed significant antibacterial activities (MIC < 100 *μ*g/mL) on 14.29% of the microorganisms studied. This is enough evidence which highlights* E. chlorantha* bark as a source of antibacterial compounds.

Classification criteria for antimicrobial activity are numerous. According to Simões et al. [34], an extract is said to be antimicrobial when MIC is between 100 and 1000 *μ*g/mL. According to Aligiannis et al. [35], the activity of an extract is strong when MIC is less than 500 *μ*g/mL, moderate when MIC values ranged from 500 *μ*g/mL to 1500 *μ*g/ml, and low when greater than 1500 *μ*g/mL. The scale proposed by Kuete [36], to which the present research referred, is much more restrictive. Whatever the case, the extract has significant antimicrobial activity meaning that it could be used to effectively fight against bacterial infections. The activity could be due to the different classes of phytochemicals found, their quantities, and the possible interactions with other constituents of the extract.* Salmonella enterica* serotype paratyphi A was the most sensitive isolate, whereas isolates from* E. coli* (EC 96),* E. aerogenes* (ENT 118), and* S. aureus* (MRSA 12) were found to be less sensitive. This suggests that the antibacterial activity of* E. chlorantha* extract is extended to both Gram− bacteria and* S. aureus*. Thus, the bacterial cell wall could have no effect on the activity of this extract.

The antimicrobial properties of a plant are influenced by several factors including the extraction solvent. Atata et al. [37] showed that aqueous, methanolic, and ethanolic extracts of* E. chlorantha* barks were active on different clinical isolates with MIC values ranging from 25 to 150 mg/mL depending on the isolate and solvent used for extraction by the disk diffusion method. In the same order, Adesokan et al. [38] revealed that the aqueous bark extract of this plant has antibacterial activity against* S. aureus*,* B. subtillis*,* E. coli*,* P. aeruginosa*, and* S. typhimurium* with MIC values ranging between 25 and 105 mg/mL. These values are very high compared to those reported elsewhere. Abdulsalami et al. [39] in Nigeria demonstrated that the methanol extract of both leaves and barks from* E. chlorantha* was active on* P. aeruginosa*,* E. coli,* and* S. typhi* with MIC values ranging from 12.5 mg/mL to 25 mg/mL by macrodilution method. Atukpawu and Ozah [40] revealed that the ethanol extract of stems, barks, and leaves was active with MIC values between 1.56 and 12.5 mg/mL, while aqueous extract MIC ranged from 6.25 to 12.5 mg/mL. The variability could be attributed to solvent used, antibacterial test method, the soil, and time of collection plant part. However the case, these findings suggest that active ingredients of* E. chlorantha* barks could be best extracted by polar solvents, thus carefully highlighting a necessity for a suitable choice of appropriate solvent for plant extraction of bioactive compounds together with the experimental methods.

The partition of methanol extract of* E. chlorantha* made it possible to obtain four fractions with varying degrees of activity. The n-butanol fraction and the aqueous residue revealed best antibacterial activities compared the crude extract and other fractions. This demonstrates that the n-butanol best concentrates the active ingredients responsible for the antibacterial activity. Therefore, the active ingredients responsible for the antibacterial activity could be of a polar nature. It will therefore be important to screen the antibacterial activity of the ethanol and water extract which express best yields [41]. In addition, this result reflects the role of fractionation in the search for antimicrobial from plants. Indeed, several alternatives have been mentioned including the exploration of crude extracts, the search for active fractions derived from crude extracts, and the research of active molecules from plants [8].

The most sensitive bacteria (MIC = 32 *μ*g/mL) to n-butanol fraction were* S. enterica serotype typhi* (SAL9),* P. aeruginosa* (CIP 76110), and* E. coli* (EC 136) showing that this fraction could be used as an alternative to infections caused by these microorganisms because many cases of resistance to these germs are reported each year worldwide [42]. Furthermore, the activity of methanol extract and its n-butanol fraction obtained on some multiresistant bacteria best valorize the* E. chlorantha* bark as a potent antibacterial since numerous researches indicate constant rise of resistant bacteria strains to common use antibiotics [5].

The MBC/MIC ratio is in most cases less than or equal to 4 meaning that the bactericidal activity of the methanol extract and n-butanol fraction from* E-chlorantha* could be expected.

Natural substances, due to the diversity of chemical they contain, act on bacteria cell constituents (membrane, wall) and/or on molecular targets (Ions or protons, proteins, DNA) by several action mechanisms [43].

Several authors showed that the inhibition of bacterial cell growth is one of the mechanisms that plant extracts explain their antibacterial activity. The growth curves illustrated that methanol extract and n-butanol fraction of* E. chlorantha* stem barks inhibited the growth of all bacteria used, the lag phase being prolonged up to 18 hours when extracts were added. The inhibition observed with Gram+ bacteria was similar to Gram− bacteria showing that the cell membrane composition did not influence the antibacterial activity. Similar results were reported by Du et al. [44] and Darah et al. [27].

According to Silva and Fernandes [45], proton pumping constitutes target of choice for antimicrobial compounds. Inhibition of these pumps by plants extract demonstrates their bactericidal activity. In fact, cytoplasmic pH bacteria cells are regulated by extrusion proton via respiratory chain and potassium influx at acid pH, and cation/proton antiporter regulates the pH in alkaline states [46]. Every substance that disturbs the regulation of ATPase responsible for maintaining the homeostasis and osmotic stability ions inside the cell are considered as a target of pumps proton. In this study* E. chlorantha* methanol extract and its n-butanol fraction inhibited the proton pumping H+-ATPases of Gram+ and Gram– bacteria, meaning that proton pumping is a possible target of this plant extract. Kuete et al. [28] and Tatsadjieu et al. [47] showed that some medicinal plants of Cameroon origin explain their antibacterial activity by inhibition of proton pumping.

The loss of salt tolerance in* S. aureus* after addition of plant extract explains the capacity of extract to disturb the capacity of bacteria to exclude salt from the cell. This is associated with alterations on the cell membrane. Rhamie and his coworkers [48] demonstrated that terpenoid compounds from* Elephantopus scaber* cause loss tolerance to cell membrane and explain her membranous target. Similar activity was also reported with essential oil of* Enteromorpha linza* Linn [29].

Biofilm formation in the environment is one of the mechanisms bacteria use to resist drugs and biocides. Many plants extract explain their bactericidal activity by the inhibition of biofilm formation. In the biofilm structure, plant extracts inhibit peptidoglycan synthesis and modulating the quorum sensing a whole of gene intervening in the regulation of biofilm formation [49]. In this study, total inhibition (100%) of biofilm formation necessitated high concentration of crude extract (4 MIC). Comparing to the crude extract, the n-butanol fraction at 8 MIC inhibited only 80% of biofilm formation. The antibiofilm activity observed indicated that the antibiofilm compounds could be found in the crude methanol extract of* E. chlorantha*. Moreover, fractioning could reduce the capacity of this extract to explain the antibiofilm. Similar results were reported by Mohsenipour and Hassanshahian [31] with ethanol extract of* Thymus vulgaris* on* E. coli*.

Phytochemical screening of* E. chlorantha* stem barks revealed several compounds such as flavonoids, phenols, alkaloids, anthocyanins, steroids, quinones, saponins, tannins, and triterpenes. Their presence could explain the bactericidal mechanism of this plant extract.

## 5. Conclusion

The methanol extract of* E. chlorantha* bark has significant antibacterial activities on the microorganisms studied. This activity extends to Gram− bacteria and* S. aureus* strains and isolates. The n-butanol and aqueous residue fraction is more active compared to the crude extract. Considering all the studied action mechanisms both on Gram− and Gram+ bacteria, it appears that the methanol extract of* E. chlorantha* could have many compounds which could inhibit the cell growth by acting as inhibition of proton pump, cytoplasmic damage, and biofilm formation inhibition.

The coexistence of several modes of action expressed by the n-butanol fraction could explain its bactericidal activity.

## Figures and Tables

**Table 1:** Bacteria strains and clinical isolates feature used in the study.

Bacteria	Characteristics	References
*Gram* ^−^		

*Escherichia coli*		[19]
ATCC10536	Reference strains	
E.C 96	Clinical isolate: IPM^S^, AMC^I^, NOR^S^, CFM^S^, CRO^R^, CIP^S^, AN^I^, CHL^S^	
E.C 99	Clinical isolate: IPM^S^, AMC^I^, NOR^S^, CFM^S^, CRO^S^, CIP^S^, AN^I^, CHL^S^	
E.C 136	Clinical isolate: IPM^S^, AMC^R^, NOR^S^, CFM^S^, CRO^S^, CIP^S^, AN^S^, CHL^S^	
E.C 137	Clinical isolate: IPM^S^, AMC^S^, NOR^S^, CFM^S^, CRO^S^, CIP^S^, AN^I^, CHL^S^	
E.C 146	Clinical isolate: IPM^S^, AMC^R^, NOR^S^, CFM^S^, CRO^R^, CIP^S^, AN^I^, CHL^S^	
*Enterococcus aerogenes*		
ATCC 13048	Reference strains	
ENT 118	Clinical isolate: IPM^S^, AMC^R^, NOR^S^, CFM^S^, CRO^S^, CIP^S^, AN^S^, CHL^S^	
ENT 119	Clinical isolate: IPM^S^, AMC^I^, NOR^S^, CFM^S^, CRO^S^, CIP^S^, AN^I^, CHL^S^	
ENT 144	Clinical isolate: IPM^S^, AMC^R^, NOR^R^, CFM^S^, CRO^S^, CIP^S^, CHL^R^	
*Klebsiella pneumoniae*		
ATCC 11296	Reference strains	
KL 111	Clinical isolate: IPM^S^, AMC^I^, NOR^S^, CFM^S^, CRO^I^, CIP^S^, AN^I^, CHL^S^	
KL 128	Clinical isolate: IPM^S^, AMC^R^, NOR^S^, CFM^S^, CRO^S^, CIP^S^, AN^S^, CHL^S^	
*Salmonella enterica serovar typhi*		
ATCC 6539	Reference strains	
SAL 9	Clinical isolate: IPM^S^, AMC^R^, NOR^S^, CFM^S^, CRO^R^, CIP^S^, AN^I^, CHL^I^	
SAL 21	Clinical isolate: IPM^S^, AMC^I^, NOR^S^, CFM^R^, CRO^S^, CIP^S^, AN^S^, CHL^I^	

*Salmonella enterica serovar paratyphi A*	Clinical isolate: AM^R^, TE^R^, SXT^R^, NA^R^, CIP^S^, CHL^S^	[20]
*Salmonella enterica serovar paratyphi B*	Clinical isolate: AM^R^, TE^R^, SXT^R^, NA^R^, CIP^S^, CHL^S^	
*Salmonella enterica serovar typhimurium*	Clinical isolate: AM^R^, TE^R^, SXT^R^, NA^R^, CIP^S^, CHL^S^	

*Pseudomonas aeruginosa*		
ATCC 27853	Reference strains	
CIP 76110	Reference strains	

*Providencia stuartii*		[21]
PS 2636	Clinical MDR isolate, AcrAB-TolC	
NEA 16	Clinical MDR isolate, AcrAB-TolC	

*Gram+*		

*Staphylococcus aureus*		[22]
ATCC 25923	Reference strains	
MRSA 9	Clinical multidrug isolate: OFX^R^, FLX^R^, K^R^, E^R^, CHL^R^, IMP/CS^R^	
MRSA 11	Clinical multidrug isolate: OFX^R^, K^R^, E^R^, CIP^R^, IM/CS^R^	
MRSA 12	Clinical multidrug isolate: OFX^R^, FLX^R^, K^R^, E^R^, IM/CS^R^	
MRSA 3	Clinical multidrug isolate: OFX^R^, K^R^, E^R^, TE^R^	
MRSA 4	Clinical multidrug isolate: OFX^R^, K^R^, CHL^R^, CIP^R^	

ST 9	Clinical isolate: IPM^S^, AMC^S^, AM^S^, DO^S^, VA^S^	[19]
ST 60	Clinical isolate: IPM^S^, AMC^S^, AM^S^, DO^S^, VA^S^, E^S^	
ST 113	Clinical isolate: IPM^S^, AMC^S^, AM^S^, DO^S^, VA^S^, E^S^	
ST 118	Clinical isolate: IPM^S^, AMC^S^, AM^S^, DO^S^, VA^S^, E^S^	
ST 120	Clinical isolate: IPM^S^, AMC^S^, AM^S^, DO^S^, VA^S^, E^S^	
ST 130	Clinical isolate: IPM^S^, AMC^S^, AM^S^, DO^S^, VA^S^, E^S^	

AN: amikacin, AM: ampicillin, AMC: amoxicillin-clavulanate, CHL: chloramphenicol, CFM: cefixime, CIP: ciprofloxacin, CRO: ceftriaxone, DO: doxycyclin, E: erythromycin, FLX: flomoxef, IMP: imipenem, IM/CS: imipenem/cilastatin sodium, K: kanamycin, NA: nalidixic acid, NOR: norfloxacin, OFX: ofloxacin, TE: tetracycline, SXT: trimethoprim-sulfamethoxazole, VA: vancomycin. R: resistant, S: sensible, and I: intermediate.

**Table 2:** Effect of fractioning of *E. chlorantha* methanol extract on MICs/MBCs (*μ*g/mL) values.

Bacteria	Extract	n-Hexane	Ethyl acetate	n-Butanol	Residual	CHL
*E. coli*						
ATCC 10536	128/256	512/-	256/1024	128/256	128/512	4/64
EC 136	128/512	512/-	256/512	32/256	64/512	32/256
EC 137	128/256	1024/-	256/1024	64/256	64/512	64/-
EC 99	128/256	1024/-	128/512	64/256	64/512	32/128
EC 96	512/1024	512/-	256/-	128/256	256/512	32/128
EC 146	64/128	512/-	1024/-	128/256	512/1024	32/256
*E. aerogenes*						
ATCC 13048	128/256	256/-	512/-	256/512	256/256	8/128
ENT 119	64/1024	256/1024	256/1024	64/512	128/512	16/128
ENT 118	512/1024	1024/-	256/-	128/1024	256/1024	128/-
ENT 144	128/256	1024/-	512/-	256/256	256/512	32/64
*K. pneumoniae*						
ATCC 11296	128/512	512/-	256/-	128/512	128/512	8/64
KL 128	128/512	1024/-	256/-	64/256	32/256	32/128
KL 111	128/512	1024/-	256/1024	128/512	256/512	32/-
*S. enterica serovar paratyphi A*	32/256	128/512	128/512	64/256	256/1024	16/64
*S. enterica serovar paratyphi B*	128/512	256/-	64/1024	64/128	128/1024	8/128
*S. enterica serovar typhimurium*	128/-	-/-	-/-	256/-	512/-	32/128
*S. enterica serovar typhi*						
ATCC 6539	128/512	512/1024	256/-	64/256	128/1024	64/256
SAL 9	128/512	512/1024	512/-	32/512	128/1024	32/128
SAL 21	256/512	512/-	256/-	128/512	128/-	64/-
*P. aeruginosa*						
ATCC 27853	128/512	256/-	1024/-	128/512	128/512	16/128
CIP 76110	128/1024	256/-	64/-	32/256	32/256	4/32
*P. stuartii*						
PS 2636	128/1024	-/-	-/-	256/-	512/-	32/-
NEA 16	64/-	-/-	-/-	256/-	256/-	8/256
*S. aureus*						
ATCC 25923	256/512	512/-	256/512	128/256	128/512	32/128
ST 120	64/512	-/-	512/512	64/256	64/512	8/64
ST 113	128/-	1024/-	256/-	64/512	128/-	16/64
ST 9	256/1024	512/-	256/-	128/256	128/-	32128
ST 118	256/1024	1024/-	256/-	128/1024	256/512	64/128
ST 130	128/512	512/-	128/1024	128/1024	128/1024	4/64
ST 60	256/1024	1024/-	128/-	64/512	64/-	8/128
MRSA 9	256/512	1024/-	1024/-	128/512	512/-	128/-
MRSA 11	256/1024	512/-	512/-	128/512	512/1024	32/128
MRSA 12	512/1024	1024/-	512/-	128/1024	256/-	64/64
MRSA 4	256/-	-/-	-/-	256/-	256/-	8/64
MRSA 3	256/-	-/-	-/-	256/-	512/-	32/128

CHL = chloramphenicol, - = MIC or MBC was greater than 1024.

**Table 3:** Extraction yields and phytochemical composition of methanol extract and fractions from *E. chlorantha *barks.

Phytochemical groups	Crude extract (7.91%)	Fractions			
		FH (5.66%)	FAE (12.5%)	FBU (20.87%)	FR (39.71%)
Alkaloids	+	−	+	+	+
Anthocyanins	−	−	−	−	−
Flavonoids	+	−	+	+	+
Phenols	+	−	−	+	+
Quinones	+	−	+	+	+
Saponins	−	−	−	−	−
Tannins	+	−	−	+	+
Sterols	+	−	+	+	+
Triterpenes	+	+	+	+	−

+: presence; −: absence, FH: n-hexane fraction, FAE: ethyl acetate fraction, FBU: n-butanol fraction, and FR: residual fraction.
